# The development, implementation and early learnings of a training program to advance interest in behavioral research careers among undergraduate BIPOC students majoring in psychology

**DOI:** 10.1186/s12909-023-04104-8

**Published:** 2023-03-15

**Authors:** Michelle R. Lent, Denise Gaither-Hardy, Kevin E. Favor, Diana Harris, Travis A. Cos, Conor Millard, Zatio Kone, Ashley Van Riper, Karen L. Dugosh

**Affiliations:** 1 School of Professional and Applied Psychology, Philadelphia College of Osteopathic Medicine, Philadelphia, PA USA; 2 Psychology and Human Services Department, Lincoln University, Lincoln University, USA; 3 Research & Evaluation Group, Public Health Management Corporation, Philadelphia, USA; 4 Department of Psychology, La Salle University, Philadelphia, USA

**Keywords:** Clinical research training program, Undergraduates, BIPOC

## Abstract

**Objectives:**

Black, indigenous and people of color (BIPOC) remain underrepresented in research occupations. This report discusses a collaboration to train undergraduate BIPOC students in clinical research between a public health institute, two medical schools, and a historically Black College or University (HBCU). This nine-month program trained BIPOC undergraduates in research methodology, psychology, and addiction science, and immersed trainees in real-world research. The program included didactic seminars, experiential activities, and a mentored research project culminating in a poster and oral presentation.

**Methods:**

Key learnings, program satisfaction survey results, and preliminary outcomes from the first three program cohorts (N = 6 students) are presented. This program addressed several barriers hypothesized to contribute to the limited number of BIPOC students pursuing research careers, including mentorship from BIPOC faculty and financial concerns.

**Results:**

Students reported moderate to high satisfaction with the program and endorsed gaining new research skills. Limitations and future directions are discussed.

**Conclusion:**

The expansion of the BIPOC health and research workforce is an urgent priority given the importance of BIPOC professionals to the health of our nation.

**Trial registration:**

ClinicalTrials.gov Identifier: NCT04650386.

## Introduction

The 1985 U.S. Department of Health and Human Services (DHHS) Task Force report on Black and minority health was heralded as a landmark document [1, 2]. The task force members, including then Secretary of DHHS Margaret Heckler, and 18 DHHS scientists, recognized that the context of healthcare disparities was consequent to complex interactions between psychological, physiological, societal, and cultural factors [1]. Task force members also recognized that the phenomena hindering equity in U.S. healthcare were poorly understood. For example, despite evidence of a narrowing of disparities in health services utilization consequent to the passage of Medicare and Medicaid, poorer access to quality care and preventive services, and concerns regarding adequate insurance coverage, persisted. Secretary Heckler, herself, characterized the persistence of such disparities overall as, “…an affront to both our ideals and to the ongoing genius of American medicine” (p. ix) [1]. The task force report identified health disparities that accounted for more than 60,000 excess deaths for racial/ethnic minority groups when compared to Whites across several conditions, including chemical dependency. Recommendations based on this report fell into six broad categories to help prioritize social-health equity for racial/ethnic minority groups. Notably, recommendations within two categories, health professions development and research agenda, underscored the importance of undertaking strategic activities for the inclusion of Black, indigenous and people of color (BIPOC) in the broadening of culturally appropriate service provision and research engagement.

More than 30 years later, however, racial and ethnic disparities are still prevalent in many critical areas [3–5] and the necessity of welcoming and preparing BIPOC students into healthcare, health policy, clinical research, and behavioral science fields remains an urgent priority. A joint report authored by the National Science Foundation (NSF) [6] and the National Center for Science and Engineering Statistics (NCSES) detailed continued, though improving, disparities in the representation of most minorities within the fields of science and engineering. According to this report, in 2019 underrepresented U.S. minority students received, 24.0% of all bachelor’s degrees and 22.1% of all master’s degrees awarded in science and engineering fields, and only 13.6% of all science and engineering doctoral degrees [6–8].

Minority Serving Institutions (MSIs) are central to the diversification of post-baccalaureate degree attainment. High Hispanic Enrollment (HHE) institutions graduated 49.2% of Hispanic/Latino bachelor’s science or engineering degree recipients in 2018 and accounted for 37.8% of Hispanic/Latinos who earned doctorates in science and engineering between 2015 and 2019 [6]. Similarly, Historically Black Colleges and Universities (HBCUs) prepared 23.2% of the African American/Black science and engineering doctorate recipients between 2015 and 2019 [6]. However, underrepresented minority doctorate holders represented only 8.9% of academic positions in 2019, far below minority representations in national demographics [6].

While MSIs represent one essential pathway to fostering greater participation of BIPOC students into research fields [9] system-level research collaborations are needed to stimulate earlier and greater interest in research careers among BIPOC students. Specifically, there is a need to establish strategic partnerships between academic institutions and research entities to foster interest in, increase access to, and prepare BIPOC students for careers as health researchers. To be maximally impactful, these opportunities should be available and accessible at each stage of students’ academic trajectories, including early in the college experience, and particularly before decisions regarding academic concentrations are formally declared.

In 2019, we received an award to conduct a randomized, controlled trial (RCT) entitled, “Enhancing office-based buprenorphine treatment: An adaptive psychosocial approach.” This project established a multidisciplinary team of investigators across four partnering institutions (an HBCU, two medical schools, and a public health institute with a research division) to achieve four specific study aims. The first three study aims focused on intervention development, study implementation, and dissemination of findings from the clinical trial. The fourth study aim, the focus of this paper, leveraged these trial activities as well as the larger strategic research partnership to develop and implement a nine-month training program for select HBCU undergraduate psychology students interested in exploring a career in addictions or psychology research. Two students rotate through the training program annually, and a total of four undergraduate cohorts will participate in the program over the duration of this four-year research initiative (N = 8). The current paper focuses on the development of this training program and key learnings from the first three cohorts of student trainees.

## Methods

At the center of this award was a four-year RCT evaluating the efficacy of an adaptive approach to providing psychosocial interventions in addiction treatment (ClinicalTrials.gov Identifier: NCT04650386). The overall goal of the RCT was to address important knowledge gaps in how to best provide psychosocial treatment to patients who are receiving office-based buprenorphine treatment for opioid use disorder (OUD) in Federally Qualified Health Center (FQHC) settings. This RCT examined the efficacy of an adaptive treatment approach, which, as the name suggests, *adapts* two psychosocial interventions (cognitive-behavioral therapy or peer support services) in accordance with the individual responses of patients to these approaches relative to treatment as usual. This adaptive approach recognized that there is no “one size fits all” treatment for OUD and that patients who are receiving medication for OUD may require different types of psychosocial support given their current and historical clinical characteristics. The primary study outcomes examined in the study were opioid abstinence and retention in buprenorphine treatment. Over half of the study sample were BIPOC (53% Black, 4% Hispanic or Puerto Rican, 2% Native American or Pacific Islander). The trial protocol was approved by the public health organization’s institutional review board (#1908). Trial participants provided written informed consent to participate.

### Research training program partnership structure

**Fig. 1:**
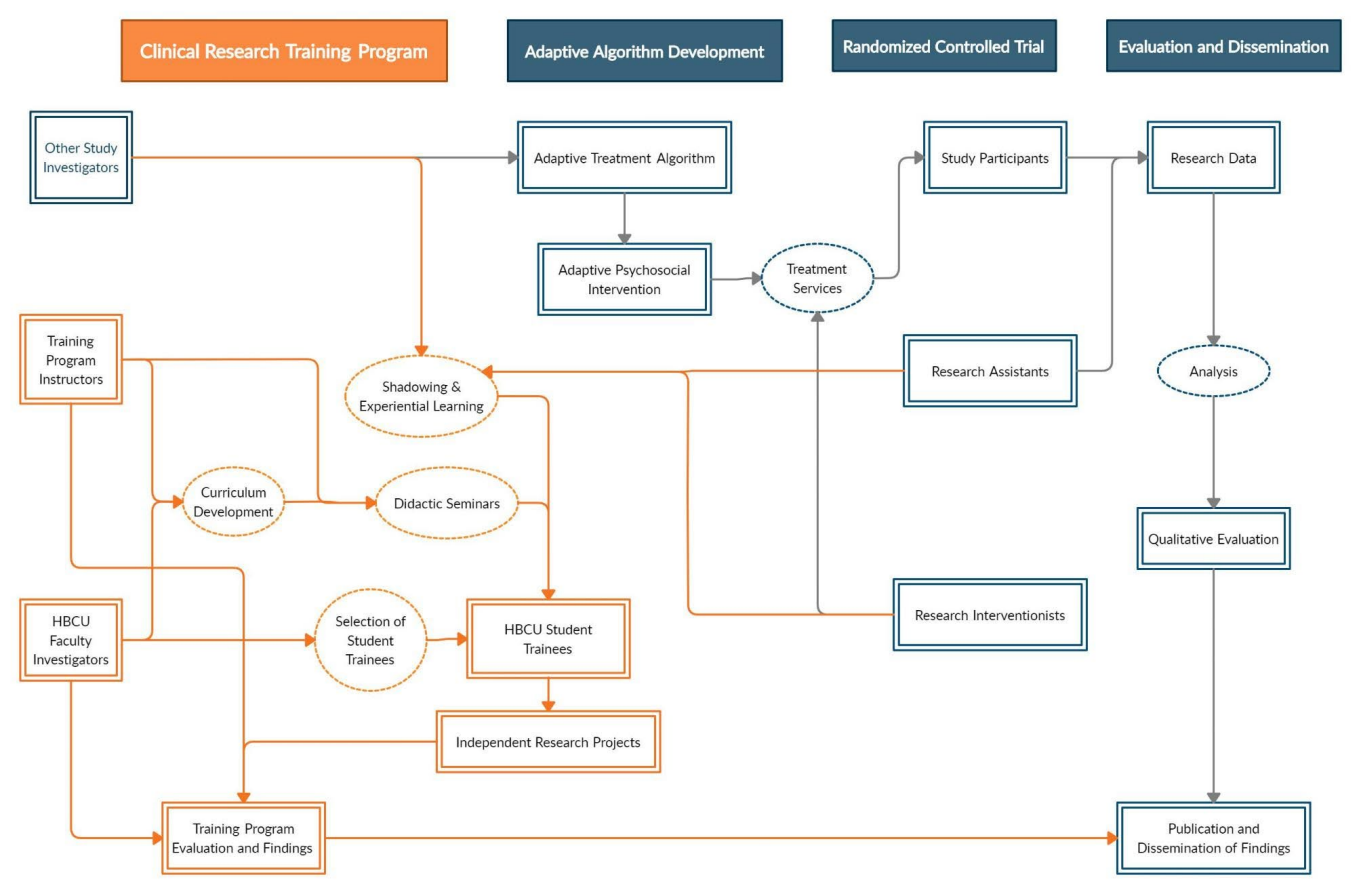
Clinical research traineeship components and their relationship to study activities

The partnership structure for this strategic research training program is multi-disciplinary and, as mentioned, multi-sectoral in nature (i.e., including three partner institution sectors - public health institute, medical schools, HBCU), with two scientific investigators representing each institution. Figure 1 illustrates the training program leadership, structure and relationship to study-related activities. Through ongoing collaboration, communication, and planning, the leadership team designed the program to feature content from a diverse set of disciplines including clinical research, addiction science, public health ethics, and psychology. The leadership team meets monthly to identify appropriate content that aligns with study-related activities and the HBCU’s academic curriculum and calendar, as well as to discuss student progress and consider modifications needed to metrics for trainee evaluation.

### Research training program objectives and components

This program is designed to train four cohorts of two undergraduate trainees annually in research methodology, psychology, and addiction science. The program immerses student trainees in real-world research activities conducted as part of the larger OUD-related RCT that illuminate the foundational research and clinical topics discussed. Specifically, this experiential training program features didactic seminars, individual research projects, and shadowing experiences that align with the overarching study’s research questions. Finally, program components help trainees to gain the skills, confidence, and experience necessary to become more competitive applicants to graduate programs. The program’s objectives are to:

1. Enhance undergraduate student interest in and understanding of clinical research;
2. Expand students’ knowledge of substance use disorders and their treatment;
3. Understand and recognize cultural and diversity considerations and their impact on marginalized communities in clinical research and treatment; and.
4. Advance student professional development and preparation for graduate-level study in addiction and psychology research, science, or practitioner-scholar career fields.

### Student selection and expectations

Annually, two undergraduate trainees are selected by the psychology faculty co-investigators from the HBCU in advance of the fall academic semester. Students are eligible if they are at least in their sophomore year of study, intending to pursue behavioral health-related academic concentrations, and able to dedicate the time required for participation (approximately 16–20 h per month including seminars, shadowing experiences, readings, and research project activities). Trainees are selected based on academic performance and relevant coursework (e.g., research methods, statistics). Trainees are provided materials (textbooks, additional readings, software), reimbursed for travel expenses, and compensated with a stipend. No course credits are offered for participation.

### Didactic seminars and readings

To achieve program objectives, comprehensive instruction on research methods and applied substance use research is provided on an academic calendar schedule via one-hour, weekly online didactic lectures and seminar discussions. Two-three instructors lead the didactics. Monthly teleconferences between the HBCU and additional training-program faculty help to maximize learning and unify messaging and communication to trainees and the larger study research team. Didactic seminars begin with an introduction to substance use disorders and the larger trial’s design and activities, and expand into topics that span foundational research methodology, research ethics, and addiction treatment approaches and their empirical support. Additional seminar topic areas are included in Table 1.

**Table 1:** Seminar Topic Areas

Seminar Topics	Shadowing, Workshops, Presentations
• Introduction to Opioid Use Disorder and the Research Study • Research Ethics and Compliance Training • The Belmont Report and Informed Consent • Database-Oriented Research and Research Question Generation • Variables and Measurement • Hypotheses and Constructs • Quantitative Research Methods • Randomized Controlled Trials and Comparative Effectiveness Research • Introduction to the Study Database • Combining Research & Practice: Scientist-practitioner model, Evidence-informed practice • Cognitive-behavioral approaches to substance use disorders • Sampling and Bias in Research Designs • How to Effectively Recruit Participants • Dissemination Principles and Strategies • Applied Program Evaluation in Substance Use • Community Informed Research • How to write a Discussion Section • How to Build a Career as a Researcher/ Psychologist	Shadowing • Research Assistant Q&A • Clinicians and providers Q&A • Community Advisory Board meeting • What is it like to be a research participant? • Discussion with a CAB member about opioid use and recovery Workshops • Creating Poster Presentations • Variable Selection and Hypotheses • Informed Consent for Clinical Trials • Data Analysis • Poster and Project Working Sessions • Poster Presentations Dress Rehearsal Presentations • Virtual poster presentations on research project findings

### Shadowing

Immersive shadowing opportunities correspond with topics discussed in the didactic seminars (Table 1).

Student trainees engage with research assistants in question and answer sessions related to consenting study participants and collecting study outcome measures, as well as informal discussions with the clinicians delivering the study’s psychosocial treatments (behavioral health counselors, peer support specialists). Additionally, the program provides trainees with structured opportunities to meet a variety of research staff members of differing backgrounds and professional expertise to help cultivate informal mentors should the trainees decide to pursue graduate study. Trainees also attend stakeholder and patient advisory board meetings for this and related research projects that are being conducted by the research team. These additional experiential activities are intended to provide students with the opportunity to see how the voices and perspectives of relevant stakeholders and communities inform the research endeavor. Additionally, shadowing provides an opportunity for trainees to better understand the research enterprise as a whole, as well as the different roles, responsibilities, and career trajectories within the research enterprise.

### Mentored research project

Trainees are provided access to existing data sets from real-world studies examining the efficacy of interventions for individuals who have substance use disorders. Faculty across the partner institutions provide intensive mentoring and instruction for student trainees to develop appropriate questions and conduct data analyses. This applied component combines instructional and experiential learning with the goal of generating and testing a research question of interest to each intern. Trainees gain experience selecting variables, developing hypotheses, preparing data, applying appropriate analytic strategies, and interpreting data. Finally, the program concludes with successful completion of a research poster at a research conference and/or presentation of their findings to a large audience comprised of faculty and staff from each partner institution. While the first two cohorts of students worked with data collected from an evaluation of the use of peer support specialists for patients with substance use disorders [10], the third cohort utilized data from the OUD RCT to answer research questions about trauma history and treatment response among in Black patients, as well as health-related quality of life in individuals with OUD.

## Results

Students who participated expressed an intent to pursue doctoral degrees post-graduation and to date, one student has enrolled in a doctoral program in psychology. As most of the remaining cohort students (n = 4) are still completing their undergraduate studies, we will continue to monitor their future career plans.

Program success was informally assessed through student self-reflections on their learning regarding research methodology and addiction treatment mid-year. Additionally, HBCU faculty regularly inquired in relevant classes regarding the students’ understanding of key concepts as they related to training program objectives.

Students also completed surveys to assess their satisfaction with the training program. Surveys consisted of 37 questions that were open-ended or asked students to rate the item (e.g., “the syllabus was clear and easy to follow”) on a five-point Likert-type scale (“Strongly Disagree” to “Strongly Agree”) or rank their favorite activities. All students strongly agreed that they were satisfied with their experience and endorsed gaining skills in quantitative and qualitative research methodology. All students also strongly agreed or agreed that they gained greater confidence in their ability to present research findings following completion of the final research project, and noted that the program content was relevant to their university coursework. The shadowing experiences most frequently ranked as students’ favorites were meetings with patient advisory boards, meetings with research assistants, and meetings with research staff providing the clinical interventions. Finally, one student indicated that they faced challenges balancing the time commitment required for full-time undergraduate study while completing the program. Greater transparency regarding the time commitment required for this program will be incorporated during the student selection phase for future cohorts.

## Discussion

This article describes an undergraduate training program in addictions research that represents an interdisciplinary partnership between an HBCU, a public health institute, and academic medical institutions. This approach represents an influential model for training and preparing undergraduate students attending HBCUs or MSIs for behavioral research careers. The program is designed to address the barriers (e.g., lack of access to mentors and peers in their field of study of the same race, economic pressures) hypothesized to contribute to the limited number of underrepresented minority students pursuing health [8, 11] and related research careers.

Thus far, the collective research team (faculty and staff across institutions) have successfully established a collaborative and supportive mentoring environment to nurture and expand trainees’ interest in behavioral research careers and ultimately grow the BIPOC scientist and scientist-practitioner workforce. Training program faculty and staff encouraged students to engage actively and frequently, and tailored their mentorship to meet the individual academic and developmental levels of each trainee, as well as their individual career goals. The pace of didactic instruction and the specific guidance provided on their research projects were determined by the individual trainees’ knowledge levels, interests, and needs. Faculty and staff modeled an adaptable, team-based approach to all aspects of the research process that fostered problem-solving and teamwork, while minimizing competition. Additionally, the program employed a “cohort” model in that two trainees participated simultaneously to promote peer mentorship and peer support among the trainees. Future programs may develop more peer mentorship activities or even establish peer support groups among current cohorts and training program graduates, which has been implemented in other types of training programs [12].

The availability of underrepresented minority mentors may influence the pursuit of doctoral study among BIPOC students and continues to be an important factor in early career-related decision-making [13]. Interdisciplinary program faculty, clinicians and staff represented a range of races, ethnicities, specialties, and backgrounds. Program faculty had expertise in a range of disciplines, including research methods, addiction medicine, behavioral health, experimental psychology, and ethics. The partnership with faculty from an HBCU was particularly important for the trainees, as they provide mentoring from Black psychology faculty that complimented the real-world research experience. The diversity of faculty and collaborators provided trainees with a range of sources from which they received individualized attention and reinforcement.

In the present program, social-health equity focused didactic content was highlighted in readings and seminar discussions, such as the historical ethical violations conducted with racial and religious minority patients, prisoners, and research participants (e.g., Nazi medical experiments, Tuskegee study). Waitzkin and colleagues [12] described a junior faculty and graduate student minority mentorship program in mental health services research, and emphasized the importance of accounting for “challenges and tensions that affect mentees’ careers and personal lives, including the emotional legacy of discrimination and historical trauma**”** (p. 205). During our training program, related topics were introduced in the context of foundational research ethics, but also to encourage understanding and exploration of trainees’ attitudes and beliefs about the U.S. healthcare system and research enterprise.

Our training objectives were similar to goals described by Glover and colleagues [14] who developed a fellowship comprised of six HBCUs and a research university. Glover and colleagues’ program for Black high school through graduate students aimed to develop students’ health disparities research and public health leadership skills through experiential learning opportunities. The authors note, however, that taking this “pipeline” approach to training requires “intensive financial and human resource investments” to be successful [14]. Programs such as the National Institute on Drug Abuse (NIDA)’s Summer Research Internship may help to answer this call by offering underrepresented high schools students experience in substance use research [15, 16].

Considering poverty and existing inequities such as structural racism, the skyrocketing costs of higher education may necessitate the need for undergraduates to sustain employment while pursuing their degrees, particularly if students are from lower socioeconomic backgrounds. For many students from lower socioeconomic households, participating in unpaid internships or training programs may be challenging, if not impossible. Moreover, the costs associated with participating in training programs such as transportation expenses and purchasing appropriate professional attire may make such opportunities prohibitive for many students. To ease these potential burdens and offer access to this program to any interested and qualified student, this program allocates annual stipends for each student, as well as additional funds for transportation costs and materials. Future programs could consider offering students the choice between a receiving a stipend or course credit to further alleviate potential obstacles to participation (e.g., financial or competing academic demands). There is a great need to establish more collaborations between academia, public health organizations, and HBCUs to best leverage each organization’s resources and assets to share and minimize costs associated with training programs.

Several limitations and key learnings are noteworthy. First, at the time of press, only three cohorts of students had completed this training program, with an additional cohort of two students expected over the next year. Overall, the total number of trainees (N = 8) is relatively small, limiting generalizability. Prior to the COVID-19 pandemic, research posters resulting from the mentored project were expected to be submitted for consideration at local or national conferences; however, the cohorts of trainees instead presented their posters via an online forum attended by more than 30 faculty and staff from the partner institutions, including leaders of these organizations. More formal measurement of success during the shadowing experiences was not assessed; however, attendance and active participation by trainees was monitored.

Notably, the first cohort of trainees were able to shadow research faculty and staff in-person for the first half of their experience because of the onset of COVID-19; all subsequent cohorts shadowed virtually. Future cohorts could consider submitting manuscripts based on their research projects for peer-review, which would further distinguish them within the graduate school applicant pool. Finally, we gathered formal feedback from students at the conclusion of the training program rather than on an ongoing basis. This, unfortunately, precluded us from altering program intensity, which may have benefitted the students throughout the duration of the program. Therefore, we recommend repeatedly obtaining feedback from students, as well as all participating staff and faculty, throughout the duration of each training year. Post-program survey responses may have been subject to a social desirability bias given that students had not yet completed their degrees and their undergraduate psychology faculty participated in the training program didactics and the mentored research project. Additionally, incorporating students into the early phases of study planning and development could allow trainees to more meaningfully contribute to the overall study team and trial.

Though all of the partner institutions were located in the Mid-Atlantic region, this training model has the potential to be replicated and expanded, and to be successfully implemented with partner organizations that are not in close geographic proximity. Through videoconferencing platforms, training program faculty were able to deliver the didactic content but also offer the trainees meaningful online experiential activities that mirrored traditional, in-person experiences. For example, trainees virtually shadowed many study-related activities including research assistant activities, meetings with interventionists and clinicians, and attendance at advisory board meetings. The innovative ways developed to conduct research and training during the COVID-19 pandemic will likely impact workflows and learning for the foreseeable future. Leveraging these learnings may allow for the development of future experiences that are not limited by the geographic locations of the students or researchers, creating greater opportunities for equity in program participation locally and nationally. Although our program intervened at the university level, future programs could intervene earlier in students’ academic trajectories. For instance, developing research training programs that target students from minority-serving middle or high schools could serve to address the “broken pipeline” earlier in students’ academic careers.

## Conclusion

In conclusion, this partnership provides one potential way to address the ongoing disparity in BIPOC representation in behavioral and addictions research. There are many paths to achieving more representative, accessible, and welcoming fields of behavioral science research for BIPOC students. Initiatives to sponsor the development of new BIPOC researchers have the potential to unlock generations of novel research approaches, experiential knowledge, and scientific innovations.

## Data Availability

No datasets are publicly available because this is a manuscript describing an undergraduate health research training program. Data are however available from the corresponding author on reasonable request.

## List of abbreviations

DHHS: Department of Health and Human Services
BIPOC: Black, Indigenous people of color
NSF: National Science Foundation
NCES: National Center for Science and Engineering Statistics
MSI: Minority Serving Institutions
HHE: High Hispanic Enrollment
HBCU: Historically Black College or University
RCT: Randomized, controlled trial
OUD: Opioid use disorder
FQHC: Federally Qualified Health Center
NIDA: National Institute on Drug Abuse
